# The complex role of genetic background in shaping the effects of spontaneous and induced mutations

**DOI:** 10.1002/yea.3530

**Published:** 2020-12-14

**Authors:** Ilan Goldstein, Ian M. Ehrenreich

**Affiliations:** ^1^ Molecular and Computational Biology Section, Department of Biological Sciences University of Southern California Los Angeles California 90089‐2910 USA

## Abstract

Spontaneous and induced mutations frequently show different phenotypic effects across genetically distinct individuals. It is generally appreciated that these background effects mainly result from genetic interactions between the mutations and segregating loci. However, the architectures and molecular bases of these genetic interactions are not well understood. Recent work in a number of model organisms has tried to advance knowledge of background effects both by using large‐scale screens to find mutations that exhibit this phenomenon and by identifying the specific loci that are involved. Here, we review this body of research, emphasizing in particular the insights it provides into both the prevalence of background effects across different mutations and the mechanisms that cause these background effects.

## Take Aways

- A large fraction of mutations show different effects in distinct individuals.
- These background effects are mainly caused by epistasis with segregating loci.
- Mapping studies show a diversity of genetic architectures can be involved.
- Genetically complex changes in gene expression are often, but not always, causative.

## Graphical abstract

Spontaneous and induced mutations can show different phenotypic effects in genetically distinct individuals (or ‘background effects’). Here, we review current work aimed at generally understanding this phenomenon.

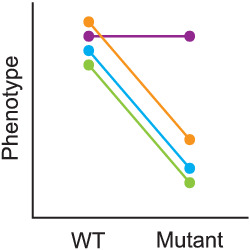

## 1 INTRODUCTION

Spontaneous and induced mutations are central to practical and theoretical problems in genetics, evolution and human health. However, the effect of a given mutation may vary depending on the rest of the genome in which it occurs. Such background effects are widespread in biology, having been found across many mutations, species and traits (Chandler, Chari, & Dworkin, 2013; Cooper, Krawczak, Polychronakos, Tyler‐Smith, & Kehrer‐Sawatzki, 2013; Nadeau, 2001). These background effects are important because they can significantly impact the relationship between individuals genotypes and phenotypes (Geiler‐Samerotte, Zhu, Goulet, Hall, & Siegal, 2016; Jarosz & Lindquist, 2010; Queitsch, Sangster, & Lindquist, 2002; Taylor & Ehrenreich, 2015b). This can have direct consequences for the total phenotypic variation within a population (Bergman & Siegal, 2003; Masel, 2013; Rutherford & Lindquist, 1998), the evolutionary trajectories of adaptive and deleterious mutations (Hemani, Knott, & Haley, 2013; Johnson, Martsul, Kryazhimskiy, & Desai, 2019; Kryazhimskiy, Rice, Jerison, & Desai, 2014), and the phenotypic prediction and therapeutic treatment of individuals carrying particular mutations (Chen et al., 2016; Narasimhan et al., 2016; Riordan & Nadeau, 2017).

Despite their prevalence and biological importance, the genetic and molecular mechanisms that cause mutations to show background effects are not fully understood. Assuming a constant environment, background effects must mainly result from genetic interactions (or epistasis) (Mullis, Matsui, Schell, Foree, & Ehrenreich, 2018). When a mutation shows a background effect, the epistasis is between the mutation and one or more unknown loci (or modifiers) that segregate within a population (Nadeau, 2001; Riordan & Nadeau, 2017). These loci may interact not only with a mutation but also each other (Chandler, Chari, Tack, & Dworkin, 2014; Dowell et al., 2010; Taylor & Ehrenreich, 2014). At the molecular level, these complex genetic interactions may arise for many reasons, which range from a mutation and epistatic loci acting in a common functional process, such as a pathway, signalling cascade, regulatory network or protein complex, to them participating in entirely unrelated processes (Kuzmin et al., 2018, 2020; Lee, Coradini, Shen, & Ehrenreich, 2019; Taylor & Ehrenreich, 2015a; Taylor, Phan, Lee, McCadden, & Ehrenreich, 2016).

**FIGURE 1:**
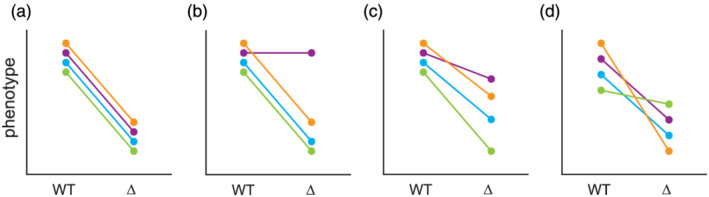
Examples of background effects. Background effects show a variety of manifestations, some of which are included here. For simplicity, we focus on haploid individuals in which a gene is either present (WT) or absent (∆). Each genetically distinct individual is illustrated using two coloured points, which denote its phenotype in both the WT and ∆ states. A given individual's response to a mutation is represented by the line connecting its two dots. These plots are intended to show how background effects can be seen at both the levels of individuals and populations. (a) No background effect: Each individual genotype shows the same response to a mutation. (b) Incomplete penetrance: Some individuals show the same response to the mutation, whereas one individual exhibits no response. (c) Variable expressivity: Individuals that respond to the mutation do so in a quantitatively different manner. (d) Line crossing: Different responses to the mutations occur, but there is no overall change in total phenotypic variation. Contrasting (b) and (c), in which total phenotypic variation changes when the mutation is present or absent, against (d) shows how mutations may or may not affect total phenotypic variation among examined individuals [Colour figure can be viewed at wileyonlinelibrary.com]

In line with their diversity of underlying genetic and molecular mechanisms, background effects can exhibit a variety of manifestations at the levels of both individuals and populations (Figure 1). Some mutations may only show effects in certain individuals (i.e., ‘incomplete penetrance’; Figure 1b), whereas others may exhibit quantitatively different effects across individuals (i.e., ‘variable expressivity’; Figure 1c) (Griffiths, Miller, Suzuki, Lewontin, & Gelbart, 2000). Often these concepts of penetrance and expressivity are used to discuss the degree of expression of a disease or some other qualitative trait among individuals who carry a particular mutation. However, it is critical to recognize that populations typically segregate for myriad quantitative traits (Mackay, Stone, & Ayroles, 2009) and the expression of these phenotypes can also be affected by the introduction of mutations (Gibson & Dworkin, 2004; Paaby & Rockman, 2014). This has led to recognition that background effects can also involve mutations increasing or decreasing the total phenotypic variation in a population (Bergman & Siegal, 2003; Jarosz & Lindquist, 2010; Queitsch, Sangster, & Lindquist, 2002; Rutherford & Lindquist, 1998) or simply altering genotype–phenotype relationships without affecting total phenotypic variation (Geiler‐Samerotte, Zhu, Goulet, Hall, & Siegal, 2016; Schell, Mullis, & Ehrenreich, 2016) (Figure 1b–d).

The above discussion highlights general awareness that epistasis between mutations and segregating loci can significantly impact the phenotypes of individuals and populations. Yet, it also belies the reality that much of this knowledge remains phenomenological and superficial. In recent years, there has been substantial effort to achieve a deeper understanding of background effects, using some of the powerful genetic tools available in model organisms. Here, we review this body of work. In particular, we synthesize research focused on identifying specific mutations that show background effects, mapping the loci that genetically interact with these mutations and determining the genetic architecture and molecular mechanisms that are responsible. This work has significantly advanced knowledge of the prevalence of background effects across mutations, as well as the genetic and molecular mechanisms that are typically involved.

## 2 HOW OFTEN DO MUTATIONS SHOW BACKGROUND EFFECTS?

Although there has long been awareness that mutations can show background effects, until recently most known cases were found due to chance or by introducing individual mutations, with known effects in reference strains, into other genetic backgrounds (Chandler, Chari, & Dworkin, 2013; Nadeau, 2001). These anecdotal studies established background effects as a phenomenon that can have major phenotypic consequences but did not clarify its prevalence across different mutations. Recent studies have attempted to provide clarity on this problem by quantitatively estimating the prevalence of background effects across different induced mutations using large‐scale screens. In this work, deletion mutations or RNA interference (RNAi) was used to disrupt a number of genes in multiple naturally occurring genotypes of the same species (Chari & Dworkin, 2013; Dowell et al., 2010; Galardini et al., 2019; Johnson, Martsul, Kryazhimskiy, & Desai, 2019; Mullis, Matsui, Schell, Foree, & Ehrenreich, 2018; Paaby et al., 2015; Vu et al., 2015). By doing this, the effects of many different mutations could then be compared across genetically distinct strains, making it possible to determine how often these mutations show background effects.

The first large‐scale screen for background effects was performed in the budding yeast *Saccharomyces cerevisiae* (Dowell et al., 2010). It focused on identifying genes that differ in their essentiality between two haploid strains; 5,100 genes were individually deleted from both strains and then examined for their effects on viability. Among these genes, 894 were found to be essential in both strains, whereas 57 were found to be essential in only one or the other. Although the vast majority of genes affecting viability were essential in both strains, 6% of these essential genes had a strain‐specific impact. The finding that essentiality can be strain‐dependent suggested that background effects may be fairly common across different genes within the same organism. However, this study's qualitative focus on gene essentiality and its analysis of only two strains likely led to underestimation of the prevalence of mutations that show background effects.

To achieve a more general understanding of this prevalence, recent studies in yeast have examined the quantitative effects of null mutations on growth across strains and environments. One study created 47 different mutant versions of the same haploid cross of two strains, each of which had a particular chromatin regulator deleted (Mullis, Matsui, Schell, Foree, & Ehrenreich, 2018). Comparison of these mutant populations with a haploid wild‐type population enabled the identification of seven deletions (15% of the total examined genes) that showed background effects. Another study examined 3,786 gene deletions in four haploid strains and 38 environments (Galardini et al., 2019). This experiment estimated that 19% of all phenotypic effects associated with gene deletions differ between strains. In yet another case, 710 known null mutations were introduced into a panel of haploid cross progeny (Johnson, Martsul, Kryazhimskiy, & Desai, 2019). Fitness of these mutant segregants and their wild‐type progenitors was then measured in a single environment. In this study, evidence suggested as many as 32% of the examined mutations showed background effects.

These prevalence estimates from yeast are supported by similar work in the fruit fly *Drosophila melanogaster* and the nematode worm *Caenorhabditis elegans*. In *D. melanogaster*, 723 hemizygous segmental deletions were examined in two different strains (Chari & Dworkin, 2013); 146 of these deletions (20%) showed different qualitative or quantitative effects on wing morphology between the two backgrounds. Additionally, two studies in *C. elegans* employed RNAi to individually perturb a number of specific genes. They then tested for qualitative and quantitative differences in the effects of these perturbations between strains. One focused on 1,353 different genes in two strains (Vu et al., 2015). In this experiment, 247 genes (18%) were found to have some difference in effect on development or reproduction between the strains. The second *C. elegans* study examined 29 genes involved in embryonic development in 55 distinct isolates (Paaby et al., 2015). All 29 genes showed evidence of different effects on development across the isolates, though it must be noted that this gene set was highly curated. A caveat to both *C. elegans* studies is that RNAi efficacy varies among strains and this could lead to artefacts in the identification of background effects (Pollard & Rockman, 2013). However, in these projects, the authors controlled for this issue during the experiments and data analyses (Paaby et al., 2015; Vu et al., 2015), suggesting prevalence estimates from *C. elegans*, are likely accurate for this organism.

Excluding the study focused specifically on *C. elegans* developmental regulators (Paaby et al., 2015), the aforementioned studies collectively suggest that 15%–32% of induced mutations show background effects. Because these insights stem from several different species, large numbers of genes, different types of genetic perturbations, and a mix of naturally occurring strains and genotypes produced in the lab by crossing, they are likely to be general and may even be underestimates. Supporting this latter point, these estimates include the entire sets of loss‐of‐function mutations examined in a given screen, many of which did not exhibit detectable effects under the conditions of these experiments. Focusing only on mutations that showed any measurable effect produces prevalence estimates as high as 74%–89%. This indicates that most loss‐of‐function mutations that show an effect in a given condition will do so in a manner that varies across backgrounds. A potential caveat to these findings is that many, but not all, of these studies were performed using a small number of genotypes. However, the already high prevalence estimates seen in these studies imply that examination of more strains would again, if anything, only lead to even higher estimates.

Another important caveat to the above estimates is that they are entirely based on strong loss‐of‐function genetic perturbations, if not nulls. Thus, comprehensive analyses of mutations that perturb gene function in a more graded manner, such as *cis* regulatory, missense and synonymous variants, are needed (Chandler et al., 2017). Such studies will be challenging to perform systematically throughout a genome because of issues with scale. For example, in *S. cerevisiae*, which has a small genome for a eukaryote, there exist ~3^12,000,000^ potential single nucleotide changes, as opposed to only ~6,000 possible single gene deletions. With this said, studies focused on small numbers of genes may help establish a more nuanced understanding of the prevalence of background effects across weaker genetic perturbations. For example, work in yeast examined the phenotypes of 5,184 genotypes containing between one and 10 nucleotide substitutions in a single tRNA gene in one haploid strain (Domingo, Diss, & Lehner, 2018). This work found pervasive evidence for single nucleotide changes showing different effects depending on the tRNA background in which they occurred, though we caution that this study may provide limited insight into background effects between mutations and segregating polymorphisms in different genes. Thus, further work is needed on the extent of background effects among more subtle genetic perturbations.

## 3 DETERMINING THE MECHANISMS THAT CAUSE BACKGROUND EFFECTS

Identifying mutations that show background effects is only a first step in more generally understanding this phenomenon. The next step is to determine the underlying genetic architecture and molecular mechanisms that are responsible (Mackay, 2014; Mackay, Stone, & Ayroles, 2009). This involves identifying the involved segregating loci and determining how they genetically interact with the mutation and each other. If resolved to the level of genes and nucleotides, such information can then be used to also investigate the molecular mechanism(s) that cause particular background effects. Detailed knowledge of genetic architecture and molecular mechanism is essential for moving beyond phenomenological explanations of background effects to a level of understanding that may eventually facilitate the prediction of background effects based on individuals' genotypes.

**FIGURE 2:**
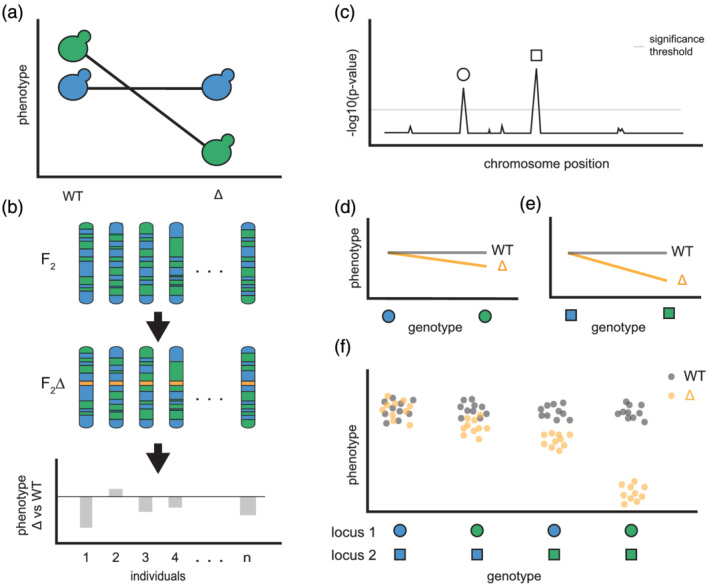
Genetic dissection of background effects. Here, we show a general experimental and data analysis workflow that could be used to determine the genetic architecture underlying a background effect. (a) A mutation in haploid yeast negatively affects the green strain but has no effect on the blue strain. (b) A cross of blue and green strains yields haploid *F*_2_ segregants. The mutation of interest (represented by a gold bar) is then introduced into each genotype, and the effect of the mutation on each genotype is measured. (c) Linkage mapping of response to the mutation identifies two loci that genetically interact with the mutation, which are denoted by the circle and square symbols. In (d) and (e), the genetic interactions between the mutation and each involved locus are examined. Individuals carrying the green allele for either locus show a decrease in phenotype when the mutation is present, whereas individuals with the blue allele show no change. (f) In this example, higher‐order epistasis between the mutation and the two loci causes the background effect. Here, neither locus contributes to phenotypic variation among wild‐type segregants. Each point in (f) represents a different segregant [Colour figure can be viewed at wileyonlinelibrary.com]

Potentially any genetic mapping approach, including linkage (Mullis, Matsui, Schell, Foree, & Ehrenreich, 2018), association (Paaby et al., 2015) or selective genotyping (Chandler, Chari, Tack, & Dworkin, 2014; Lee, Coradini, Shen, & Ehrenreich, 2019; Lee, Taylor, Shen, & Ehrenreich, 2016; Taylor & Ehrenreich, 2014, 2015b; Taylor, Phan, Lee, McCadden, & Ehrenreich, 2016), can be used to identify individual loci that contribute to background effects. Relative to conventional quantitative trait locus mapping, the genetic dissection of background effects simply involves an extra step of introducing mutations of interest into genetically diverse individuals (Mullis, Matsui, Schell, Foree, & Ehrenreich, 2018; Paaby et al., 2015). These individuals' phenotypic responses to the mutations can then be measured and loci influencing these responses mapped (Figure 2). However, in many cases, understanding the architecture of background effects requires being able to identify complex genetic interactions between a mutation and multiple specific loci (e.g., Chandler, Chari, Tack, & Dworkin, 2014; Hou, Tan, Fink, Andrews, & Boone, 2019; Lee, Coradini, Shen, & Ehrenreich, 2019; Lee, Taylor, Shen, & Ehrenreich, 2016; Taylor & Ehrenreich, 2014; Taylor & Ehrenreich, 2015b; Taylor, Phan, Lee, McCadden, & Ehrenreich, 2016). Approaches that utilize crosses, rather than naturally occurring genotypes, may perform better for teasing apart such complex epistasis because they minimize confounding by population structure, as well as variance in single and multilocus genotype frequencies (Ehrenreich, 2017; Forsberg, Bloom, Sadhu, Kruglyak, & Carlborg, 2017; Taylor & Ehrenreich, 2015a).

**FIGURE 3:**
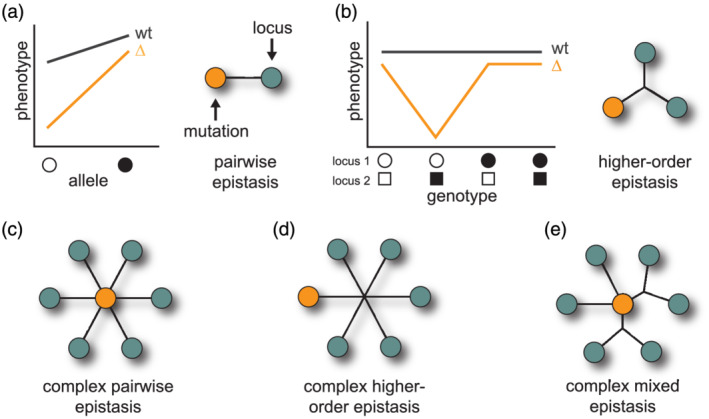
Forms of epistasis underlying background effects. Background effects arise because of epistasis between mutations and segregating loci. These genetic interactions can vary in the number of involved loci, as well as the contribution of higher‐order epistasis between a mutation and multiple loci. In (a) and (b), hypothetical genotype–phenotype relationships are shown for a pairwise genetic interaction between a mutation and a single locus and for a higher‐order genetic interaction between a mutation and two loci, respectively. Example graphical representations of these interactions are shown for each case. In this and subsequent higher‐order genetic interactions, the intersections of black lines are used to signify epistasis between a combination of more than two loci. In (c)–(e), different architectures of epistasis between mutations and loci are shown. (c) displays a mutation exhibiting a number of pairwise genetic interactions with loci. In contrast, (d) shows a mutation exhibiting higher‐order epistasis with many loci. (e) illustrates how a background effect could involve a mix of pairwise and higher‐order epistasis between a mutation and loci [Colour figure can be viewed at wileyonlinelibrary.com]

After loci that genetically interact with a mutation have been identified, the nature of their interactions with a mutation and each other can be examined in more detail (Figure 3). Specifically, such data can be used to address a number of questions, including the following: How many loci contribute to a given background effect? How do the phenotypic contributions of involved loci differ when a mutation is present or absent? What are the forms of genetic interactions between a mutation and loci that produce a given background effect? Moving beyond these more statistical questions requires identifying the exact genes and genetic variants involved. Achieving this level of resolution may require additional fine mapping using crosses or genome editing. Of course, advances in CRISPR/Cas9 technologies (Cong et al., 2013; Mali et al., 2013), including the development of highly parallelized genome editing (Roy et al., 2018; Sadhu et al., 2018; Sharon et al., 2018), will likely accelerate such work. Techniques like these will aid in systematically determining the specific genes and nucleotides participating in background effects, which is a critical step in generally addressing key questions about the molecular mechanisms by which mutations and loci genetically interact.

## 4 EMPIRICAL INSIGHTS INTO THE GENETIC UNDERPINNINGS OF BACKGROUND EFFECTS

Genetic mapping of examples in which mutations show background effects has begun to answer the questions posed in the last section. Regarding numbers of contributing loci, several studies indicate that background effects can involve large numbers of loci. Arguably, the first work to suggest this was on the essential protein chaperone Hsp90. Perturbation of Hsp90 produces substantial, background‐dependent phenotypic changes in *D. melanogaster* (Rutherford & Lindquist, 1998)*,* the thale cress *Arabidoposis thaliana* (Queitsch, Sangster, & Lindquist, 2002)*,* yeast (Geiler‐Samerotte, Zhu, Goulet, Hall, & Siegal, 2016; Jarosz & Lindquist, 2010) and potentially even humans (Karras, Yi, D'andrea, Whitesell, & Lindquist Correspondence, 2017). To get at the genetic bases of these responses, linkage mapping was performed in a yeast cross grown in a number of environments (Jarosz & Lindquist, 2010). This identified 107 loci that exhibit diverse responses to Hsp90 perturbation. In response to Hsp90 perturbation, some of these loci had effects that toggled on (or off) in a binary manner, although others had effects that were quantitatively modified. This study estimated that as many as 20% of the genetic variants genome‐wide show epistasis with Hsp90, though this has yet to be explicitly proven.

More recent research supports a highly polygenic basis for certain background effects. One line of evidence comes from the application of genomic heritability analysis to differences in response to particular mutations among genetically distinct individuals. Genomic heritability approaches estimate the collective contribution of all common genetic variants within a population to phenotype (de los Campos, Sorensen, & Gianola, 2015; Meuwissen, Hayes, & Goddard, 2001; Yang et al., 2010; Yang, Zeng, Goddard, Wray, & Visscher, 2017). Within the context of mutations showing background effects, the trait examined in a genomic heritability analysis is the difference in phenotype between the wild‐type and mutant versions of each included genotype. Genomic heritability has a range between zero and one, and high genomic heritability estimates would imply that response to a given mutation is controlled by many loci throughout the genome. This is exactly what was found for most of the genes in the *C. elegans* RNAi study of developmental regulators described earlier (Paaby et al., 2015). Specifically, response to perturbation of 19 out of 29 examined developmental regulators (66%) showed genomic heritability measurements that were greater than 0.6, suggesting that response to those perturbations was influenced by a large number of loci.

The limitation of genomic heritability analysis is that specific contributing loci are not identified. For background effects involving many loci, mapping these loci is technically challenging because doing so requires large mapping populations in which a mutation either segregates or can be introduced into all individuals. Such a study was performed in *S. cerevisiae* and found more than a thousand loci that genetically interacted with seven different mutations (Mullis, Matsui, Schell, Foree, & Ehrenreich, 2018). These loci were detected on every chromosome and in nearly all chromosomal windows, showing that loci with the potential to contribute to background effects are in fact common throughout the genome. This finding was similar to what had previously been shown for Hsp90 (Jarosz & Lindquist, 2010), but with an order of magnitude more loci detected, likely due to the use of a mapping population that was roughly an order of magnitude larger.

To this point, we have emphasized background effects involving a large number of loci, which by definition must individually make small contributions. However, there are many cases in which this model does not hold and instead background effects involve smaller numbers of loci that collectively produce large effects despite showing no measurable effect on their own. This was initially suggested by the aforementioned work on strain‐specific gene essentiality in yeast, which found evidence that most cases of conditional essentiality were caused by three or more loci interacting with mutations and each other (Dowell et al., 2010). Although the involved loci were not identified in this initial work, shortly thereafter, subsequent studies in both *Drosophila* and yeast provided clear proof of such complex epistasis playing a major role in background effects (Chandler, Chari, Tack, & Dworkin, 2014; Hou, Tan, Fink, Andrews, & Boone, 2019; Lee, Coradini, Shen, & Ehrenreich, 2019; Lee, Taylor, Shen, & Ehrenreich, 2016; Taylor & Ehrenreich, 2014; Taylor & Ehrenreich, 2015b; Taylor, Phan, Lee, McCadden, & Ehrenreich, 2016). These findings illustrate the diverse epistatic architectures that can give rise to background effects (Figure 3).

## 5 MOLECULAR MECHANISMS UNDERLYING BACKGROUND EFFECTS

Another objective in studying background effects shown by mutations is to obtain insight into their causative molecular mechanisms. Much is generally known about the mechanisms that cause epistasis (Boone, Bussey, & Andrews, 2007; Domingo, Baeza‐Centurion, & Lehner, 2019; Lehner, 2011; Phillips, 2008), but this work is largely based on the analysis of combinations of loss‐of‐function mutations generated in the lab (Costanzo et al., 2016; Kuzmin et al., 2018, 2020). Thus, to learn about the specific molecular mechanisms that cause background effects between mutations and segregating loci, it is critical to identify the specific genes and genetic variants that are involved. However, such work is difficult to scale to many loci. Also, in many cases, information on individual loci may not be sufficient to determine how multiple loci genetically interact with a mutation and each other to produce a given background effect. For these reasons, insights into the mechanisms that cause background effects come not only from the characterization of involved loci but also from more indirect approaches, such as gene expression analyses, computational simulations, and leveraging information from different deleted genes that produce similar background effects across genetically distinct individuals. In this section, we try to integrate these sources of information.

Work in both humans and *C. elegans* indicates that the effect of a mutation in a given individual will often be related to the impacted gene's expression level prior to the mutation (Hutchinson et al., 2003; Vithana et al., 2003; Vu et al., 2015). This is mainly relevant to situations in which a mutation has only a partial loss‐of‐function. In *C. elegans*, such partial loss‐of‐function can be easily generated using RNAi, which may knock down the transcript level of a given gene without completely abrogating its activity. Utilizing RNAi, one study analysed phenotypic responses to perturbation of 1,353 genes in two strains (Vu et al., 2015). In addition to the responses, genome‐wide gene expression was also measured, making it possible to relate observed background effects to pre‐existing gene expression differences between the strains. Specifically, it was found that the strain expressing a given gene at a lower level typically exhibited a more severe phenotypic response to RNAi targeting that gene. In contrast, the strain that expressed a targeted gene at a higher level showed a weaker phenotypic response, if any. These results speak to the important role of gene expression in modulating the effects of certain mutations.

In addition, both theoretical and empirical works support an important role for genetic variation in gene regulatory networks as a major influence on how different individuals will respond to the same mutations. This makes sense given that transcription of a given gene is likely to be controlled by complex networks of transcription factors and other regulatory proteins, as well as by feedback from the gene itself (Barabási & Oltvai, 2004; Davidson & Levine, 2005). Computational simulations show that complex regulatory networks have the inherent ability to reduce the effects of (or buffer) genetic variants in genes present in or regulated by the networks (Bergman & Siegal, 2003). However, this same work showed that perturbation of these regulatory networks by single gene deletions could dramatically increase the heritable phenotypic variation within populations, because of changes in the effects of alleles that had previously been buffered. Examination of many different mutations in silico suggested this ability to modulate the effects of segregating loci should be common across mutated genes, which is consistent with work on the prevalence of background effects described earlier. Indeed, empirical research in yeast, worms and flies has confirmed these theoretical expectations, identifying many cases in which mutations expose genetic differences in signalling pathways and gene regulatory networks, resulting in background effects (Chandler, Chari, Tack, & Dworkin, 2014; Lee, Coradini, Shen, & Ehrenreich, 2019; Lee, Taylor, Shen, & Ehrenreich, 2016; Matsui, Linder, Phan, Seidl, & Ehrenreich, 2015; Mullis, Matsui, Schell, Foree, & Ehrenreich, 2018; New & Lehner, 2019; Taylor & Ehrenreich, 2014, 2015b; Taylor, Phan, Lee, McCadden, & Ehrenreich, 2016; Torres Cleuren et al., 2019).

Although genetic variation in gene regulation can clearly play an important role in the manifestation of background effects, there is also ample evidence that background effects can arise in the absence of such variation. For example, analysis of background effects in the yeast cysteine biosynthesis pathway found missense, and nonsense alleles in a key enzyme within the pathway were responsible (Hou, Tan, Fink, Andrews, & Boone, 2019). In a different yeast example, background effects seen upon deletion of a lysine deacetylase did not appear to be mediated through transcription, but rather through missense polymorphisms that likely affect the function of nuclear‐encoded mitochondrial proteins (Schell, Mullis, Matsui, Foree, & Ehrenreich, 2020). In some cases, the genetic interactions between mutations and loci that cause background effects may even be influenced by genes outside the nucleus, such as in the mitochondria (Edwards, Symbor‐Nagrabska, Dollard, Gifford, & Fink, 2014). Increasingly, evidence also suggests that in some cases, background effects may result from nonspecific genetic interactions that are mediated through fitness or other global cellular features (Johnson, Martsul, Kryazhimskiy, & Desai, 2019; Kryazhimskiy, Rice, Jerison, & Desai, 2014). This stands in contrast to the other examples discussed in this section, in which mutated genes and their interacting loci had discernible functional relationships.

## 6 CONCLUSION

Recent work suggests that a substantial fraction of all mutations exhibit background effects. However, this finding is admittedly based on strong loss‐of‐function mutations, and more systematic analysis of weaker genetic perturbations is also needed. Usually, these background effects involve multiple, if not many, loci that show epistasis with a mutation. In some cases, loci that genetically interact with mutations to produce background effects also exhibit epistasis with each other. Data indicate these genetic interactions between mutations and loci frequently arise due to complex changes in gene regulation but by no means is this universal. Clear examples exist in which there are no changes in gene expression, and instead, the background effects entirely stem from missense polymorphisms that likely impact protein structure–function relationships or from epistatic relationships that may be mediated nonspecifically through global features of the cell.

Although the involvement of many loci and diverse molecular mechanisms in background effects is similar to complex traits in general (Bloom et al., 2015; Bloom, Ehrenreich, Loo, Lite, & Kruglyak, 2013; Hallin et al., 2016; Linder, Seidl, Ha, & Ehrenreich, 2016; She & Jarosz, 2018), a key difference is the significance of epistasis. With notable exceptions (Shao et al., 2008; Yazbek et al., 2011; Pavlicev, Norgard, Fawcett, & Cheverud, 2011; Spiezio, Takada, Shiroishi, & Nadeau, 2012; W. Huang et al., 2012; Shorter et al., 2015; Forsberg, Bloom, Sadhu, Kruglyak, & Carlborg, 2017; Zan & Carlborg, 2020), a large body of research suggests that complex trait variation in the absence of mutations like those discussed in this paper has a mainly additive genetic basis (Bloom et al., 2015; Bloom, Ehrenreich, Loo, Lite, & Kruglyak, 2013; Hallin et al., 2016; Hill, Goddard, & Visscher, 2008; Yang et al., 2010). In contrast, epistasis is of central importance in determining how individuals respond to mutations (Hou, Tan, Fink, Andrews, & Boone, 2019; Mullis, Matsui, Schell, Foree, & Ehrenreich, 2018). When background effects occur, it means that introduced mutations have caused alleles at other loci to show different effects. Thus, a valid, alternative interpretation of background effects is as situations in which mutations modify the genetic architecture of impacted traits through their genetic interactions with segregating loci (Jarosz, Taipale, & Lindquist, 2010; Schell, Mullis, & Ehrenreich, 2016).

**FIGURE 4:**
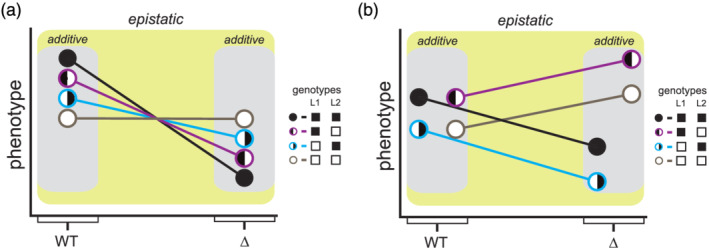
The role of epistasis in background effects is not inconsistent with the importance of additivity in conventional studies of complex traits. In this figure, we provide simple examples of how segregating loci may act additively in the presence or absence of a mutation but may show epistasis when the mutant and wild‐type individuals are combined together. For both plots, L1 and L2 denote two different loci that segregate within a population. The circles represent the expected mean for a particular two‐locus genotype class, with the genotype of the class indicated using black or white colouring inside the circle. In (a), L1 and L2 each have effects in both the presence and absence of the mutation. For both loci, the black allele produces higher phenotypic values when the mutation is absent, but lower phenotypic values when the mutation is absent. The change in the effects of these loci between mutant and wild‐type individuals represents epistasis between the mutation and loci. In (b), the effect of L1 remains the same in the presence of the mutation. In contrast, L2 has no effect among wild‐type individuals despite having an effect among mutants. Thus, introduction of the mutation leads to a significant change in the expected phenotypes of the different genotype classes [Colour figure can be viewed at wileyonlinelibrary.com]

Why these two bodies of work—background effect and conventional complex trait studies—produce distinct insights about epistasis is both intriguing and unresolved. A potential biological explanation is that mutations showing background effects have different properties than the natural genetic variants that tend to persist within populations over time. This could be because the mutations have not gone through the sieve of natural selection or may perturb molecular systems to a greater degree. Yet, it is also possible that these contrasting findings have a technical basis. Conventional genetic mapping studies may be ill suited to detect complex epistasis among segregating loci, even if it is present (Carlborg & Haley, 2004; Cordell, 2009; Ehrenreich, 2017; Mackay, 2014; Taylor & Ehrenreich, 2015a). There are several potential reasons for this, such as statistical power limitations and population structure confounding. However, it is important to note that regardless of why these types of studies typically produce different findings, they remain compatible. For example, as shown in Figure 4, loci could act in a predominantly additive manner in the presence or absence of a mutation but show genetic interactions with a mutation when both mutant and wild‐type individuals are considered at the same time.

In conclusion, the findings discussed in this review suggest that the importance of these background effects to traits of human interest could be greater than presently appreciated. For example, many clinical phenotypes, including neurological disorders (Gauthier & Rouleau, 2012; Y. Huang, Yu, Wu, & Tang, 2014), hereditary cancers (Bogaert & Prenen, 2014) and other diseases (Acuna‐Hidalgo, Veltman, & Hoischen, 2016; Veltman & Brunner, 2012), have been found to involve both mutations and segregating loci. Despite significant effort, the genetic basis of these disorders is only partially known, and it is entirely plausible that this could be due to the fact that these mutations and loci genetically interact in complicated ways that have not yet been detected. Of course, the challenge is that in humans and many other species, it will be difficult to identify and fully determine the causes of any such background effects. Thus, model organism research, such as the work reviewed in this paper, will likely remain critical for advancing fundamental understanding of the mechanisms that cause mutations to show background effects.
